# Epidemiology of Cardiovascular Diseases in Morocco: A Systematic Review

**DOI:** 10.1900/RDS.2021.17.57

**Published:** 2021-10-31

**Authors:** Rida Elyamani, Abdelmajid Soulaymani, Hind Hami

**Affiliations:** Laboratory of Genetics and Biometry. Faculty of Science. Ibn Tofail University. Kenitra. Morocco.

**Keywords:** cardiovascular disease, epidemiology, Morocco, heart, Africa

## Abstract

**OBJECTIVE:**

To provide a systematic review of studies on cardiovascular diseases (CVD) and their risk factors in the Moroccan population.

**METHODS:**

A systematic analysis was performed based on PRISMA guidelines by retrieving data bases (Medline, Embase, and other) using technical keywords in addition to manual research on official websites. Published studies in the English or French language, conducted in Morocco or concerning the Moroccan population within the last two decades, were identified.

**RESULTS:**

This is the first systematic review of CVD in Morocco. Data from 159 studies were retrieved and analyzed. Most studies were written in the English language (75.89%) and published between 2010 and 2019 (85.47%). The mortality rate caused by CVD in Morocco has reached 38%, with ischemic heart disease and stroke as the main events causing death (31.0% and 22.5% respectively). The risk factors present in the population studied were headed by tobacco smoking (45-50%), followed by physical inactivity (21.1%), elevated rate of hypertension (25.3%), and depression (5.47%). Impacted by a high rate of illiteracy and poverty and an unprepared health care system in Morocco, these numbers are expected to increase over the next decade.

**CONCLUSIONS:**

Based on these alarming incidences, investment in scientific research and epidemiological studies should be increased to determine the needs of the local population. The available evidence shows that the risk of cardiovascular disease and the associated mortality is very high in Morocco and will rise in the next years prospectively, which calls for urgent multi-sectorial approaches and treatment strategies.

## 1 Introduction

The global prevalence of diseases has increased drastically over the last three decades. Leading causes of disease and morbidity have shifted from communicable causes with infectious, maternal, and perinatal diseases to non-communicable diseases (NCD) and cardiovascular diseases (CVD) [1]. This epidemiological trend may be explained by the theory of epidemiological transition, whereby the general socioeconomic progress of a country will cause a health status shift from communicable diseases to NCD [2, 3].

In 2016, the World Health Organization (WHO) estimated a global mortality of 17.9 million deaths caused by CVD, representing 31% of total deaths. When all cases of heart disease were considered, ischemic heart disease (IHD) was seen as the leading cause of death, with an estimated 7.29 million people dying from acute myocardial infraction (95% CI: 6.8-7.81) and almost 110.55 million prevalent cases, followed by stroke and ischemic stroke, which caused the second and third largest number of cases of heart disorders to result in disability-adjusted life years (DALYS). It has been estimated that there were 5.39 million acute first-ever ischemic stroke cases (95% CI: 5.02-5.73), 3.58 million cases of acute first-ever hemorrhagic and other forms of stroke (95% CI: 3.34-3.82), and 42.43 million prevalent cases of cerebrovascular diseases (95% CI: 42.07-42.77) overall in 2015. These phenomena are not equally distributed among countries. Instead, they depend on several factors such as culture, risk factor distribution, ethnicity (genetics), economy, and geographical location [4].

There is a relationship between CVD mortality and socioeconomic index (SEI) in societies. It increases sharply as SEI (SEI > 0.25) increases, shifting from women to men, and in high income countries (SEI > 0.75) it decreases again [5]. This is explained by technological progress and pharmacological advancements [6] associated with increasing awareness and improvement in health care access among populations of high-income countries. In contrast, there is a high mortality caused by CVD (80%) in low- and middle-income countries (LMIC) [7], but epidemiological investigations into CVD and the prevalence of its risk factors are few, particularly in rural or poor urban areas. It has been estimated that CVD data were unavailable in almost 89.8% of Sub-Saharan countries and in 48.1% of northern African countries compared to only 0.3% of high-income countries [5]. Therefore, the need for more studies in this research area is urgent, as is the need to devote resources to healthcare systems and interventional programs, which would allow scientists to evaluate the effectiveness of preventive programs and healthcare policy-makers to address new strategies depending on the needs of the local populations.

In this review, we provide a comprehensive systematic analysis of published studies in both the French and English language, and we included data collected in the Moroccan population over the last 20 years, in an effort to provide a reference work on the status of CVD in Morocco.

## 2 Methods

### 2.1 Search strategy

**Figure 1.**
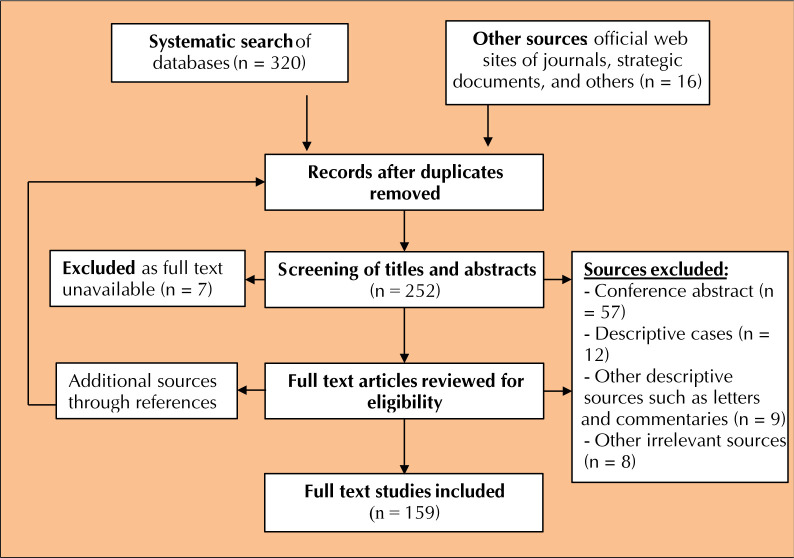
Flowchart of study selection following the PRISMA protocol.

This systematic review was performed according to the PRISMA protocol. **Figure 1** shows the flowchart for selecting studies for this review. An electronic systemic retrieval was applied in the following databases: Embase, Medline, Epub (Ovid), Cochrane Central, Web of Science, Science Direct, PubMed, and Google Scholar. Additionally, a manual search in the following journals and official websites was performed: Nature, New England Journal of Medicine, WHO, Ministry of Health in Morocco, and others.

The search was carried out using specific keywords in the English and French language by combining 2 technical keywords referring to our topic. The first level of keywords included: “cardiovascular diseases”, “cardiology”, “heart”, “vascular”, “circulatory”, “angina”, “stroke”, “myocardial infraction”, “cerebrovascular diseases”, “arrhythmia”, “aneurysm”, “heart failure”, etc. The second level of keywords referred to the risk factors: “hypertension”, “high blood pressure”, “diabetes”, “obesity”, “physical activity”, “smoking”, “cigarettes”, “metabolic syndrome”, “psychological stress”, “depression”, etc. These words were combined with referral keywords to define the location, e.g.: “Morocco”, “North Africa”, “Africa”, “Euro-Mediterranean region”, “Arabic countries”, “Global”, etc. The search was carried out until August 2020.

### 2.2 Data extraction

All retrieved studies that focused on cardiovascular diseases, cardiology, heart diseases, and their risk factors and that were conducted in Morocco or in the Moroccan population as part of multi-centric studies, including cross-sectional studies, case series, population-based studies, etc., were considered for inclusion in this systematic review and were extracted to consider as primary data. Secondary data on CVD risk factors were not considered.

### 2.3 Quality assessment

All sources were reviewed by all experts and scored according to their category, context, correctness, clarity, and contribution to CVD knowledge and whether they refer to the Moroccan population. Studies with low population size and genetic studies were not included in this study.

### 2.4 Data synthesis

Primary data from full text sources were extracted and entered into standardized tables containing the following columns: authors, year of publication, type of study, language, research funding, population, and outcome.

## 3 Results and discussion

### 3.1 Study characteristics

**Figure 2.**
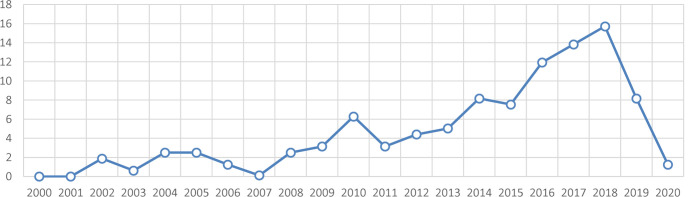
Number of publications on CVD in Morocco since the year 2000 (in %).

This study is the first systematic review of CVD in Morocco. We have identified 336 primary sources and, after filtration by exclusion criteria, 159 full text publications were included in the study. Most publications were written in the English language compared to the French language (75.89% vs. 24.11%). Most studies were published between 2010 and 2019 (**Figure 2**).

The studies were classified into 5 groups:

1. Case-control studies (12.39%)
2. Theoretical studies, review studies, reports of case series, systematic reviews, strategic documents, official documents (23.14%)
3. Cross-sectional studies (33.09%)
4. Prospective studies, longitudinal studies (13.22%)
5. Retrospective studies and data analysis (18.18%)

**Figure 3.**
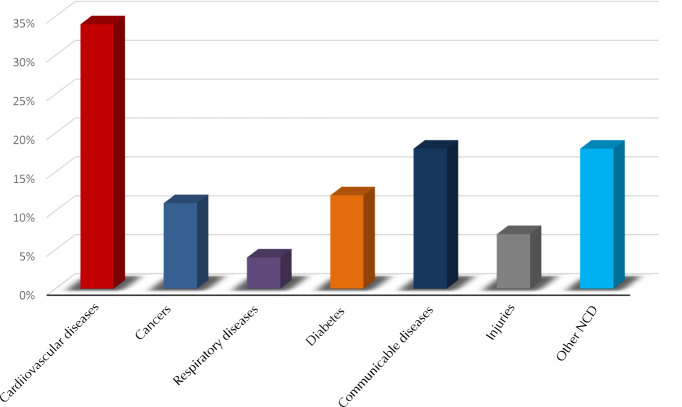
**Causes of mortality in Morocco in percentages in 2016**. According to the WHO, NCDs are estimated to be responsible for 80% of all deaths.

### 3.2 CVD mortality

In 2015, the mortality rate of CVD patients in the eastern Mediterranean region (EMR) ranked intermediately (27.7%) between Europe (46%) and Africa (12%). It is expected to rise to the second-highest position (31.1%) after Europe by 2030. In Morocco, 80% of total deaths were due to NCDs, with CVD as primary cause of death (38%) followed by cancer (18%) and chronic respiratory diseases (6%) [8]. Between 2007 and 2017, ischemic heart disease was the first cause of death (22.1%) followed by stroke (15%). During the same period, diabetes mortality progressed from the 9^th^ to the 4^th^ position (increase of 35.4%) and hypertension-related heart disease mortality from the 10^th^ to the 6^th^ position as cause of death (increase of 27.6%) [9].

### 3.4 Pathology of CVD

CVD is the generic term for diseases that affect the heart directly and includes disorders of the blood and vascular system. The conditions underlying atherosclerosis develop over a long time, occur generally at middle age or later, and include ischemic heart disease or coronary artery disease (mainly heart attack), cerebrovascular diseases, various form of stroke, peripheral diseases, arteritis, and hypertension. Also, there are other forms of CVD that are described as essential, namely congenital and rheumatic heart disease, cardiomyopathy, and arrhythmia [5].

In the EMR, the most prevalent cardiac disorders in 2015 were [10]:

- Ischemic heart disease (47%)
- Cerebrovascular disease (20%)
- Hypertension (7.2%)
- Rheumatic heart disease (4.5%)
- Inflammatory heart disease (4.1%)

A large retrospective study of 16,002 subjects from the general population followed up at the Military Hospital (Rabat) for CVD conducted between 2000 and 2016 showed that the distribution of the various cardiac pathologies in Morocco was as follows [11]:

- Hypertension (67.4%)
- Cardiomyopathy (23.2%)
- Valvulopathies (3.6%)
- Ischemic heart disease (2.2%)
- Heart failure (2.2%)
- Arterial diseases (1%)

**Figure 4.**
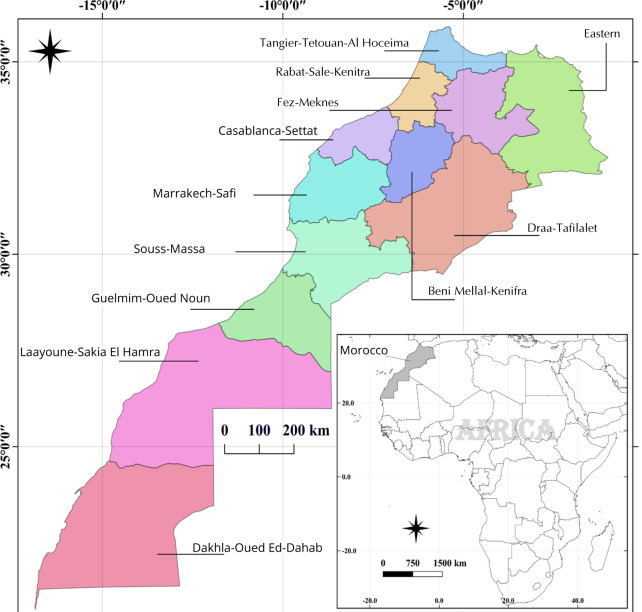
Map of the regions of Morocco (12 regions) [17].

The patients had an average disease duration of 8 ± 2.74 years, and more men than women were affected. A study of the development of heart failure among subjects with left bundle branch block showed that these subjects have a high prevalence of idiopathic dilated cardiomyopathy compared to other forms (39.1% vs. 4.8% p < 0.001), with a high frequency of hospitalization for heart failure (64.5% vs. 23.3%, p < 0.001), and that left ventricular ejection fraction was significantly lower in this group [12]. Myocardial infraction was studied in the Fes [13] and Rabat region [14], with results indicating a high percentage among men (aged 58 years and older), 51% in the Fes and 57.28% in the Rabat region, with smoking as the dominant risk factor. A large population cohort study in the region of Rabat-Casablanca conducted between 2008 and 2009 showed that rural residents had a higher risk of stroke compared with urban residents (338 vs. 252 per 100,000 persons) [15]. The three major pathological mechanisms implicated were:

1. Ischemic stroke (70.9%)
2. Intra-cerebral hemorrhage (8.7%)
3. Subarachnoid hemorrhage (3.9%)

The rest (16.5%) was not identified. Another retrospective study of stroke in the Marrakech population indicated that the major risk factors were: diabetes (41.8%), hypertension (65.45%), dyslipidemia (10.0%), and obesity (15.45%) [16].

## 4 CVD risk factors

### 4.1 Tobacco use

Tobacco is one of the most important risk factors for CVD. Globally, the prevalence of smoking was 21.9 in 2016 %; the frequency for the EMR was 19.8% and the estimated prevalence for Moroccan adults amounted to 24.0% [18]. In 2000, the national survey released by the Moroccan Ministry of Health indicated a smoking prevalence of 17.2% [19]. In 2017, the Moroccan Ministry of Health conducted a study according to the WHO standardized method for collecting, analyzing, and disseminating data on NCD risk factors in different countries, called STEPwise Approach to NCD Risk Factor Surveillance (STEPS) which indicated that the prevalence of tobacco use (all forms) was 13.45% [20]. At any time studied, men always smoked more than women (26.9% vs. 0.4%) and tobacco use was more prevalent in urban than rural areas. The highest frequencies of smoking were observed in the age groups 30-44 and 45-50 years. The average age for starting smoking was 19 years old (urban areas 19, rural areas 18.7 years old), and the average duration of smoking was 21.6 years old. The same analysis indicated that more than 60% of Moroccan tobacco users smoke an average of 10 to 24 cigarettes per day.

Multiple logistic regression [21] demonstrated that age, gender (male), marital status (divorced), occupation (low education), and region of residence (Casablanca area) were the strongest determinants of current smoking in Morocco. A retrospective analysis from 2014 in the Casablanca area showed that the risk of death attributed to cigarette smoking was 16.2% in male and 2.0% in female residents [22]. Ischemic heart disease and cerebrovascular diseases were the third and fourth leading cause of death caused by smoking.

The real prevalence of tobacco use could be higher than the announced results if we consider that in Morocco there is a social construction about female smoking which is considered as incorrect behavior even in public places. Therefore, females will always try to mask their tobacco use even in epidemiological surveys [23]. Also, the increase in other forms of tobacco usage such as water pipe-smoking, also known as “Shisha”, among young men could affect the total frequency of smoking in a population, bearing in mind that many studies do not include these types of smoking in their analysis. During the last two decades, preventive measures have been carried out by the Ministry of Health in Morocco to reduce the prevalence of smoking, essentially by creating an anti-tobacco information campaign via TV, radio, and print journals. However, a large proportion of the Moroccan population, particularly illiterate persons and subjects from rural areas, are unaware of this campaign.

### 4.2 Physical inactivity

There are no official statistics about physical inactivity in Morocco. However, the WHO estimates a higher frequency for the EMR than the rest of the world, namely 34.9% (43.5% women, 26.9% men). A large data analysis of 57,000 persons aged 25 to 64 years old from 22 African countries including Morocco was carried out between 2003 and 2009 and indicated that African populations are not more active than Asian, European, and North American populations [24]. A survey conducted in 2008 including 2,613 subjects across Morocco showed that the prevalence of low physical activity was 16.5% (24% women vs. 9% men) [25]. The results of the STEPS study (2017) indicated that the percentage of the population that did not match WHO recommendations for physical activity (i.e. 150 minutes of moderate physical activity per week) was 21.1%, while more women than men were concerned (26% vs. 16.1%) [20].

Physical inactivity increased with age, from 31.5% (60-69 years) to 49.3% (≥70 years), and was higher among urban than rural residents; only 14.2% of rural residents were found not to match WHO specifications (all forms of physical activity). The major fraction of the total amount of physical activity was related to daily jobs, and 84.7% of the general population was not physically active in their free time at all.

In Morocco, the main determinants of low physical activity among men are [25]:

- Age
- Unemployment (retirement)
- High income
- Overweight or obesity

The main determinants among women are:

- Living in urban areas
- Being a housewife

Also, physical inactivity was prevalent in secondary school, where 58.8% were inactive (50% boys and 66.6% girls). Most kids spent much of their time in front of the TV (1/3) and more time on weekdays in the sitting position than at the weekend (p < 0.001) [26]. Boys were more active than girls; they were 8 times more likely to meet the recommendation of at least 60 minutes of moderate to vigorous physical activity per day (OR: 8.569; 95% CI: 4.23-17.32, p < 0.001) [27].

### 4.3 Nutrition and alcohol consumption

Nutrition may contribute drastically to the development of CVD by acting on diabetes, hypertension, dyslipidemia, and their outcomes. According to the WHO, the EMR has one of the healthiest diets in the world, with a low rate of alcohol consumption (0.1% for subjects ≥ 15 years old) and an average intake of fruit of 130 g/day and vegetables of 200 g/day, although sodium intake is high (3.95 g/day). A Moroccan study with 2,214 subjects showed that only 29.9% had a low adherence to this diet; these were most likely to be single or divorced individuals, rural residents (OR: 1.46, 95% CI: 1.02-2.08), and obese individuals (OR: 1.56, 95% CI: 1.16-2.11) [28]. A pilot study of 132 Moroccan participants assessing salt intake by measuring urine secretion showed that 71.2% had a daily sodium intake exceeding WHO recommendations [29]. Another analysis of Moroccan fast-food consumption measuring the concentration of sodium revealed that participants consumed high values of sodium ranging from 0.25 g per 100 g in minced meat sandwiches to 1.1g per 100 g in pizza [30]. In Morocco, the chemical analysis of commercial white bread showed an average salt concentration of 17.42 ± 1.28 g per kg, which is the equivalent to a daily intake of 8 to 9 g salt through the bread alone and exceeds all recommendations [31].

Nutritional changes in Morocco are also documented by the increase in daily caloric supply per capita, from 2,410 kcal in 1968/70 to 3,031 kcal in 1997/98 [32]. The national STEPS analysis from 2017 showed that red and white meat were consumed on 4.2 days/week, eggs on 2.8 days/week, fish on 1.6 days/ week, and milk products on 4 days/week on average [20]. Almost 2/3 of the general population thought that they consumed only the necessary amount of salt. The prevalence of fast food was 7.1%, consumed more frequently by men than women (8% vs. 6.1%) and by urban than rural residents (8.8% vs. 3.9%). Surprisingly, urban residents reported that they consumed more vegetables and fruit than rural residents. The analysis of urinary salt concentration (24 hr) revealed that participants consumed 10.6 g/day (men 11.9 g/day vs. women 9.3 g/ day) on average [20].

Morocco has a low (official) rate of alcohol consumption and addiction because of religious considerations. Among the general population, 93% reported that they had never consumed alcohol in their lifetime, and the prevalence of its consumption for the last 30 days was 1.7%. This factor is more prevalent in men than women, with 99.6% of women reported to have never tasted alcohol before, and among urban compared to rural residents (2.2% vs. 0.8%). Finally, alcohol addiction was more frequent in rural than urban residents (24.3% vs. 13.3%).

### 4.4 Obesity

According to the WHO (2016), the global prevalence of overweight and obesity was 39% and 13%, respectively [33]. The EMR has the third highest prevalence of obesity in the world (23.6% women vs. 14.6% men). In Africa, obesity was more frequent among women than men (10-15% women vs. 4-5% men). In Morocco, obesity and overweight were estimated to be 21.7% (15.6% men vs. 27.6% women) and 55.1% (50.5% men vs. 59.6% women), respectively [34]. The national survey from 2000 estimated the prevalence of obesity to be 13.2% in the general population [19], and results from the STEPS study in 2017 estimated 20% prevalence of obesity and 53% prevalence of overweight, with a higher frequency among women than men (63.4% vs. 57.6%) and among urban than rural residents (57.6% vs. 44.7%) [20].

In eastern Morocco, the prevalence of overweight and obesity were 40.3% and 25.1%, respectively, with more women than men (32.7% vs. 11.5%, p < 0.001) and more urban than rural residents affected [35]. A study of 1,818 adolescents showed that the prevalence of obesity was 3.41% and was correlated with having a father (OR = 1.58, p = 0.008) or mother with higher education (OR = 1.56, p = 0.009), high family income (OR = 2.11, p = 0.028), children transported to school by car (adjusted OR = 1.77, p = 0.017), using a computer daily (>4 h/day, OR = 2.56, p = 0.004), and consuming high amounts of soft drinks and soda (OR = 1.42, p = 0.04) [36]. Another study of body perception among university students in Beni-Mellal showed that 69.8% were not satisfied with their body, and 28.6% stated that they wanted to be heavier, while the true prevalence of obesity was 11.4% [37]. In a study on obesity in children, conducted in Marrakech in 2013 (n = 1,418), the prevalence of overweight and obesity was 8% (95% CI: 6.7-9.6) and 3% (95% CI: 2.2-4.1), respectively, based on WHO references [38].

In Morocco, female predisposition to obesity is linked to motherhood and the social view of a women’s body, according to which women are considered to have an “attractive body” if they are heavier and have hip fat [39]. Therefore, it is probable that some women increase their calorie intake to gain weight. Also, Moroccan women tend to hire housemaids for housework which reduces their daily physical activity [40]. Owning private cars and the increasing consumption of a western diet play additional roles in the increasing prevalence of obesity in both genders.

### 4.5 Hypertension

Hypertension is the leading cause of CVD worldwide. In 2010, the global prevalence of hypertension was 31.1% (95% CI: 30-32.2%); it was slightly higher in men than women (31.9% vs. 30.1%) and higher in low-income and middle-income (LIMI) countries than in high-income countries (31.5% vs. 28.5%) [41]. Findings from the PURE study indicate that rural residents from LIMI countries tend to have a high prevalence of hypertension compared to industrialized countries where rural residents have a lower prevalence [42].

Morocco has one of the highest prevalence of hypertension in the EMR [34]. In 2000, the estimated prevalence was 33.7% [19]. Another large population study with 10,714 subjects from across Morocco, conducted between 2008 and 2009, revealed a total prevalence of 39.8% and an age-adjusted prevalence of 26.6%, affecting more women than men (28.0% vs. 26.3%, p = 0.01) and more rural than urban residents (26.4% vs. 22.9%, p < 0.005) [43]. In the STEPS analysis from 2017, the prevalence was estimated to be 29.3% with no difference in gender or residence status [20].

Almost 3/4 of subjects with elevated blood pressure do not follow any pharmacological treatment. It was observed that women attended blood pressure checks more regularly than men, especially in rural areas. The use of pharmacological treatment increases with age; about 5.3% of subjects already diagnosed with hypertension were under herbal treatment. A retrospective study of 2,000 hypertensive subjects indicate that age, rural residence, diabetes, elevated body mass index (BMI), waist size, and hypercholesterolemia were the main determinants of hypertension among the Moroccan population, but in that study, gender, smoking, and family history were not considered as risk factors for hypertension [44].

The prevalence of hypertension in eastern Morocco was estimated to be 31.7%, with no gender difference, but a higher frequency in rural than urban areas (39.9% vs. 29%, p < 0.001) [45]. A study conducted in the Rabat region showed that hypertension and pre-hypertension were even prevalent in adolescents aged 11-17 years (9.6% and 17.9%, respectively) [46].

### 4.6 Dyslipidemia

According to the WHO, the global prevalence of hypercholesterolemia, defined as ≥190 mg/dl cholesterol in subjects ≥25 years, was 38.9% [47]. The prevalence for the EMR was estimated to be 38.9%, and more women than men were affected (40.4% vs. 36.2%) [47]. Annually, elevated cholesterol is responsible for 2.5 million deaths and 29.7 million disabilities and contributes 1/3 of the mortality by ischemic heart disease. A 10% reduction in blood cholesterol is considered to be correlated with a 50% reduction in heart disease within 5 years [48].

In Morocco, the results of the STEPS analysis revealed that the national prevalence of hypercholesterolemia (≥190 mg/dl) was 10.5%, but only 2% of subjects were under pharmacological treatment [20]. High frequencies were recorded among women and in urban areas. The same study indicated that 7.1% of the patients had switched their treatment from prescribed pharmacological drugs to herbal or traditional treatments. The prevalence of low high-density lipoprotein (HDL) cholesterol, defined as <40 mg/dl for men and <50 mg/dl for women, was 59.3% for women and 54.3% for men. The prevalence of raised blood triglycerides, defined as ≥150 mg/dl for women and ≥180 mg/dl for men, was 15.4% for men and 9.3% for women, and was more prevalent in urban than rural areas (16.8% vs. 12.9%). Lipid testing is an expensive procedure. This may explain why a large proportion of the general population (88%) never checked their lipid status.

### 4.7 Diabetes mellitus

In 2014, the WHO estimated that 422 million persons were affected by diabetes, which translated into a global prevalence of 8.5%. Diabetes was considered directly responsible for 1.5 million deaths and indirectly responsible for 2.2 million deaths. It is more common in the EMR than Europe (13.7% vs. 7.3%) [50]. The estimated prevalence for Morocco in 2016 was 12.4%, affecting men and women almost equally (12.6% vs 12.3%) [51].

While the national survey of 2000 indicated a prevalence of 6.6% [19], the 2017 survey (STEPS) indicated a national prevalence of 10.6%, affecting more women than men (12.6% vs. 8.6%) and more urban than rural residents (12.1% vs. 8.0%) [20]. The same study indicated that 2/3 of the Moroccan population never checked their glycemic status, which applies more frequently to women than men (71.5% vs. 55.2%) and to rural than urban residents (72.4% vs. 58.2%). Approximately, 71.6% of diabetic subjects were under pharmacological treatment, and remarkably women tend to visit traditional therapists and change their treatment more frequently than men (7.1% vs. 1.6%).

**Table 1.** The prevalence of CVD risk factors

Risk factor	Year	Men	Women	Total
Premature death between 30-70 years (%)	2016	13	11	12
Harmful use of alcohol (%)	2016	1	0	1
Physical inactivity	2016	20	31	25
Salt intake (adults >20 years) (g/day)	2010	12	10	11
Tobacco use (current smoking)	2016	48	1	24
Raised blood pressure (%)	2015	24	24	24
Diabetes (%)	2014	13	12	12
Obesity (adults >18 years) (%)	2016	19	32	26

**Legend:** Risk factors classified according to the WHO [49].

Regional studies in Morocco showed various frequencies for different regions: the prevalence of diabetes among women in the Meknes region was estimated to be 19% [52], and the general prevalence in eastern Morocco to be 10.2% (10.7% in women vs. 9.3% in men) [35]. Urban areas in eastern Morocco were more affected than the rural areas (10.9% vs. 7.9%). A cross-sectional study revealed that only a few diabetes patients (0.4%) have achieved the three physiological targets (namely HbA1c <7%, systolic blood pressure <130 mmHg, diastolic blood pressure <80 mmHg, and LDL-cholesterol <1 g/l), indicating the high cardiovascular risk for Moroccan diabetics [53].

Analysis of the eating behavior of people with diabetes showed that their regime includes a high percentage of lipids (mainly monounsaturated fatty acid) and proteins [54]; while fish consumption was low, they largely consumed cold meat, cheese, green vegetables (which are part of traditional Moroccan food, in particular tajine and couscous). Another study evaluating therapeutic education showed that the vast majority of Moroccan diabetics (91%) receive education about hygienic measurements, diet, and glycemic self-control (98.0%) [55]. The same study indicated that 34% of diabetes patients do not respect this education.

The social dimensions of diabetes in Morocco are very complicated [56]. Findings show that 20.5% of diabetics hide their condition at their workplace, probably because of social competition or to avoid questions. This situation may lead to clinical complications; for example, 18.2% experienced hypoglycemia at their workplace. Morocco is an Islamic state, where, during Ramadan, when Muslims fast during the day. Moroccan diabetics are advised not to fast, but almost 58% of them fast for the entire month, nevertheless [57, 58]. This is another reason for the occurrence of diabetes complications; 10.4% experience episodes of severe hypoglycemia during this time.

### 4.8 Psychological stress

Chronic psychological stress can lead to CVD through anxiety and depression. Globally, depression and anxiety affect 264 and 284 million persons, respectively, which translates to a general prevalence of 3.4% and 3.8% [59].

**Table 2.** National health spending in 2011 [61]

National health accounts in Morocco	Value
Total public health spendin, including consumer expenditures, fiscal funding, and medical insurance (million $)	5,593
Total public health spending on cardiovascular diseases, diabetes, cancer, and chronic respiratory diseases (million $)	777
Total consumer expenditures on health (million $)	2,996
Total consumer expenditures on cardiovascular diseases, diabetes, cancer, and chronic respiratory diseases (million $)	364
Total public health spending on cardiovascular diseases, diabetes, cancer, and chronic respiratory diseases (% of total health spending)	13.9
Total consumer expenditures on cardiovascular diseases, diabetes, cancer, and chronic respiratory diseases (% of total consumer expenditures)	12.2
Consumer expenditure on CVD, diabetes, cancer, and CRD relative to total expenditure on 4 NCDs (%)	46.9

Morocco has a high rate of depression (5.47%), which affects women more frequently than men (6.37% vs. 4.34%) and causes 958.37 DALYS per 100,000 persons. The estimated prevalence of anxiety was 4.99%, affecting more females than men (6.21% vs. 3.71%) [60]. The real frequencies of these affections could be underestimated because of the lack of health system structures besides the social stigmatization attached to mental illnesses, which means that people avoid consulting for mental health problems.

## 5 Moroccan health care system and cardiovascular diseases

In Morocco, the health care system is undergoing drastic changes in order to catch up with the epidemiological processes. Composed essentially by the public and private sector [62], the system is inadequately distributed over the country, with centralization at the Atlantic coast region followed by big cities and urban areas, while the rest of Morocco experiences health staff shortages, especially physicians, specialists, and clinical pharmacists. In 2015, according to the Moroccan Ministry of Health, the inter-regional dispersion of physicians was 21% [63]. The average density of physicians in Morocco is 2.5 per 10,000 persons, which is much less than the average recorded in the EMR, which is 11.4 per 10,000 persons (WHO) [64]. These numbers reflect the gap between population needs and healthcare system capabilities.

In 2011, the total national health spending for cardiovascular diseases, diabetes, cancer, and chronic respiratory diseases represented 13.9% of the total public health spending, 96.2% of which was on curative targets and only 3.8% on preventive measures [61], which reflects a lack of strategy. Beside these conditions, the high frequency of illiteracy in the Moroccan population (26%) [65], cultural traditions, and the absence of psychological counseling may act in concert to accelerate the process of atherogenesis and increasing mortality. For example, in Moroccan primary health care centers, it is common to neglect the follow-up of patients with high cardiac risk because of a lack of guidelines to classify patients according to their risk factors and cardiac risk stages. Also, it is very common to see diabetes and hypertensive patients abandon their prescribed pharmacological treatment and seek herbal or even “spiritual” treatment offered by traditional therapists [66].

Certainly, the Moroccan pharmaceutical market in antihypertensive and antidiabetic medication has increased by the penetration of generics, but prescription patterns vary widely and do not always match WHO recommendations [67]. The high rates of poverty and unemployment, the lack of education, and long-term exposure to risk factors may act in concert to trigger chronic conditions, in particular obesity, hypertension, and diabetes. When individuals see their health gradually deteriorating this increases their psychological stress and causes episodes of depression. They also may lose trust in pharmaceutical treatment and healthcare staff who may convey their frustration at their unfavorable working conditions and shortage of medical equipment and pharmaceutics to the patients.

The governmental strategy for the prevention of NCDs for 2019-2029 in cooperation with the WHO is based on 4 levels [61]:

1. Preventive actions
2. Increasing healthcare accessibility
3. Improving health guidance
4. Expanding health surveillance

The success of this strategy may be related to effective actions, which has to start by facing the healthcare staff shortage. The Ministry of Health should increase the availability of pharmaceutical treatment and biomedical equipment in hospitals, particularly in rural and remote areas.

## 6 Conclusions

In conclusion, the Moroccan population faces a high rate of cardiovascular diseases, which is mirrored by a high rate of risk factors, with particularly elevated rates of hypertension, obesity, and depression, while the response by the healthcare system is inadequate. Available projections for the next few years show that Morocco may even expect an increase in these rates, which may cause higher mortality and disabilities. Simultaneously, the healthcare system is still unprepared and tends to disregard major health problems. Furthermore, the Moroccan population is characterized by a high rate of illiteracy and poverty as well as inequalities in the access to proper healthcare. In the face of these epidemics, it is the responsibility of the Ministry of Health to take appropriate action by following multi-sectorial strategies and approaches involving local communities, schools, universities, and other civil associations to succeed in meeting the upcoming health challenges in the next decade.

## Abbreviations

CD: communicable diseases

CI: confidence interval

CRD: chronic respiratory diseases

CVD: cardiovascular diseases

DALYS: disability adjusted life years

EMR: Euro-Mediterranean region

IHD: ischemic heart disease

LMIC: low to middle income countries

NCD: non-communicable diseases

OR: odds ratio

PA: physical activity

PRISMA: preferred reporting items for systemic reviews and meta-analysis

PURE-study: prospective urban-rural epidemiological study

SEI: socioeconomic index

WHO: World Health Organization
